# Preliminary clinical outcomes of shoelace repair with internal brace for ulnar collateral ligament injuries with chronic avulsion bone fragments in student baseball players

**DOI:** 10.1016/j.jseint.2026.101649

**Published:** 2026-01-29

**Authors:** Yasuhiro Mitsui, Kazuto Higuchi, Koji Hara, Ryunosuke Abe, Syuichiro Sakai, Toshihiko Yoshida

**Affiliations:** Hyakutake Orthopedic and Sports Clinic, Saga, Japan

**Keywords:** Ulnar collateral ligament, Internal brace, Shoelace repair, Avulsion bone fragment, Baseball players, Return to play, Student athletes

## Abstract

**Background:**

Ulnar collateral ligament (UCL) repair with internal brace augmentation has demonstrated promising results. Traditionally, chronic avulsion bone fragments (BFs) at the UCL attachment site have been considered as indications for reconstruction. We applied a shoelace repair technique combined with internal bracing in student baseball players with and without such BFs. We hypothesized that favorable clinical outcomes could be achieved regardless of the presence of BFs. This study aimed to evaluate the clinical outcomes of shoelace repair with internal brace technique.

**Methods:**

Eighteen student baseball players who underwent shoelace repair with internal brace were included. Based on pre-operative radiographs and computed tomography scans, patients were classified into those with BFs (n = 8) and those without (n = 10). Post-operative outcomes were assessed and compared between the 2 groups using Japan Orthopedic Association sports score, disabilities of the arm, shoulder, and hand sports module, Kerlan–Jobe Orthopedic Clinic score, and time to return to play (RTP).

**Results:**

All the patients returned to play. No significant differences were observed between the groups in sports scores (*P* = .73, *P* = .74, *P* = .93, respectively), time to RTP (*P* = .58), or Conway scale (*P* = .67). No complications were observed in either group.

**Conclusion:**

Shoelace repair with internal brace may be a viable surgical option for UCL injuries with chronic avulsion BFs in carefully selected young athletes with minimal ligament degeneration and good tissue quality, particularly when early RTP is prioritized.

Ulnar collateral ligament (UCL) injury is one of the most severe throwing-related injuries affecting the upper extremity. In baseball players, it typically results from cumulative microtrauma due to repetitive throwing motions.^3^^,^^9^ In the United States, the number of UCL reconstruction surgeries significantly increased from 2007 to 2011, with the highest rates observed in baseball players aged 15-19 years, showing an average annual growth of about 9%.^15^ Furthermore, by 2025, the incidence of UCL reconstruction in this age group is projected to reach 14.5 per 100,000 individuals.^7^^,^^22^

Initial treatment options include cessation of throwing, physical therapy, and conservative approaches such as platelet-rich plasma therapy^8^^,^^20^ and extracorporeal shock wave therapy.^20^ If conservative management fails, surgical intervention is considered. The standard surgical treatment for UCL injury is ligament reconstruction using a tendon graft, which has demonstrated favorable outcomes.^6^^,^^10^^,^^17^^,^^21^^,^^36^ However, a major limitation of reconstruction is the prolonged recovery time, often resulting in the loss of 1 to 2 competitive seasons.^32^

Recently, UCL repair using internal brace (IB) augmentation with FiberTape (Arthrex, Naples, FL, USA) has garnered attention.^5^^,^^14^ Dugas et al^14^ reported that IB provided superior initial fixation strength compared with reconstruction in biomechanical studies. In addition, IB demonstrated comparable resistance to gapping and failure under high valgus torque.^4^^,^^5^ In clinical studies, Dugas et al^12^ performed IB repair in overhead athletes aiming for early return to play (RTP). Reconstruction was performed in cases with bone fragments (BFs) or severe ligament degeneration. They found comparable clinical outcomes, with IB offering a shorter time to RTP.

According to Savoie et al,^31^ younger overhead athletes typically exhibit less ligament degeneration than professionals, making them more suitable candidates for UCL repair rather than reconstruction. Based on this rationale, we considered that the shoelace repair with internal brace augmentation (SRIB) could also be effective in cases involving BFs. Traditionally, these cases have been considered indications for reconstruction, based on the assumption that primary repair is not feasible after fragment removal. This technique may allow for primary repair even after fragment excision, potentially expanding the indications for UCL repair. Since January 2022, we have performed SRIB in student baseball players with minimal ligament degeneration, based on magnetic resonance imaging (MRI), ultrasound, and intraoperative findings, regardless of the presence of BFs. Previous reports have indicated that valgus instability may occur following medial epicondyle avulsion fractures, regardless of whether bony union is achieved.^16^^,^^27^ Harada et al^18^ also reported that nonunion of medial epicondyle fragments may be associated with subsequent recurrence and other injuries. Untreated childhood avulsion injuries of the medial epicondyle may result in chronic BFs, increasing the risk of medial elbow pain and UCL insufficiency during adolescence.^26^ Therefore, addressing these chronic fragments during surgical repair may help prevent long-term dysfunction and further support the indication of SRIB in such cases.

This study aimed to investigate the influence of BFs on the short-term post-operative outcomes of SRIB in student baseball players. We hypothesized that favorable clinical outcomes could be achieved regardless of the presence of BFs.

## Materials and methods

### Patient population

This study was approved by the relevant institutional review board (approval number 2502); all patients provided informed consent regarding participation and publication of the study. This study included patients diagnosed with UCL injury between January 2022 and July 2024. The inclusion criteria for UCL injury were: (1) tenderness along the UCL, (2) imaging findings on MRI or ultrasound indicating UCL injury, and (3) positive results on both the moving valgus stress test and milking test.

**Figure 1:**
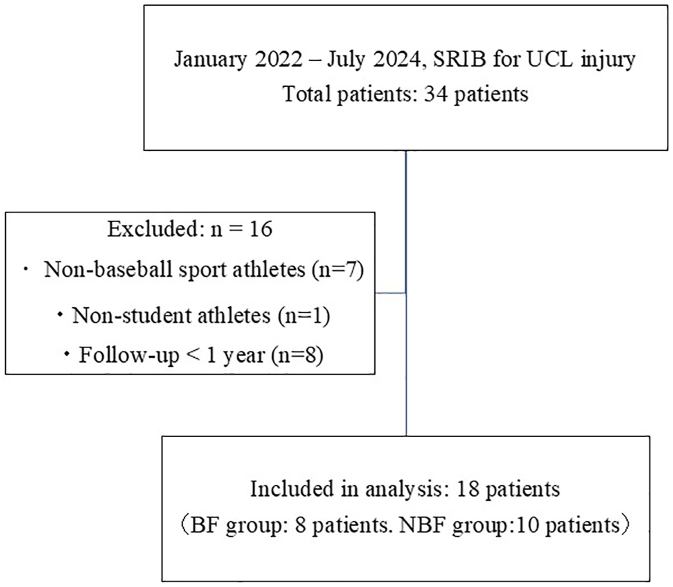
Flow diagram of patient selection. A total of 34 patients underwent SRIB for UCL injury between January 2022 and July 2024. After applying the inclusion and exclusion criteria, 18 student baseball players were included in the final analysis (8 with BFs and 10 without [NBF]). Excluded cases included adult athletes, nonbaseball players, and those with less than 1-year post-operative follow-up. *SRIB*, shoelace with internal brace; *UCL*, ulnar collateral ligament; *BF*, bone fragment; *NBF*, no bone fragment.

Surgical indications were as follows: (1) failure of conservative treatment for more than 3 months, (2) medial joint space widening of ≥2 mm in the affected elbow compared with the contralateral (uninjured) side on ultrasound performed under gravity stress (shoulder 90° abduction, elbow 90° flexion), and (3) cases requiring early return to sports. Exclusion criteria were: (1) athletes participating in sports other than baseball, (2) nonstudent (adult) athletes, and (3) patients with less than 1 year of post-operative follow-up. After applying these criteria, 18 patients were included in the final analysis (Fig. 1). Chronic avulsion BFs were defined as residual ossified fragments at the proximal or distal UCL attachment, clearly separated from the bone on radiographs or computed tomography.

### Image findings

UCL injury classification was performed using pre-operative MRI (1.5 T; Philips Medical Systems, Tokyo, Japan), based on the method described by Ramkumar et al.^28^^,^^29^ Proximal partial tears were classified as type 1A and complete tears as type 1B; midsubstance partial and complete tears as types 2A and 2B, respectively; and distal partial and complete tears as types 3A and 3B, respectively.

BFs were evaluated using pre-operative radiographs and computed tomography (Canon Medical Systems, Tokyo, Japan). Fragments located between the medial epicondyle and sublime tubercle were classified based on their relative position: those closer to the medial epicondyle were categorized as proximal, and those closer to the sublime tubercle as distal. Patients with BFs were classified as BF–positive, and those without BF as no bone fragment (NBF). All BF cases involved chronic avulsion-type BFs.

### Operative technique

**Figure 2:**
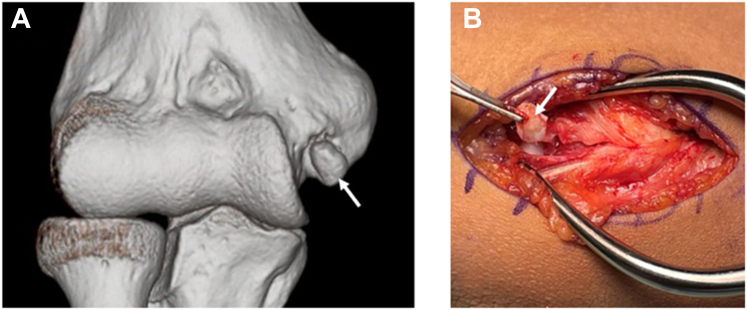
Pre-operative and intraoperative images. (**A**) Pre-operative 3D-CT image showing a chronic avulsion-type bone fragment at the proximal UCL attachment site (*white arrow*). (**B**) Intraoperative image after splitting the UCL along the direction of its fibers, showing the excised proximal bone fragment (*white arrow*). *3D*, three dimensional; *CT*, computed tomography; *UCL*, ulnar collateral ligament.

All procedures were performed by a single senior orthopedic surgeon specializing in elbow surgery (Y.M.). Surgery was performed through a muscle-splitting approach to allow direct visualization and assessment of the UCL. If significant ligament degeneration was observed intraoperatively, the procedure was converted to ligament reconstruction. One patient was converted to ligament reconstruction owing to severe ligament degeneration that was deemed unsuitable for repair; this case was excluded from the final analysis. The UCL was split along the direction of its fibers. When chronic avulsion-type BFs were present at the proximal or distal ligament attachment, they were often found embedded within the ligament substance. In our practice, SRIB is considered feasible when the avulsion BF is ≤ 1 cm; for larger fragments, ligament reconstruction is preferred. These fragments were carefully excised in a minimally invasive manner to preserve the structural integrity of the ligament. Although minor ligament defects were unavoidable due to the intraligamentous nature of the fragments, excessive resection was meticulously avoided, as a large defect could preclude the use of repair techniques (Fig. 2, *A* and *B*).

**Figure 3:**
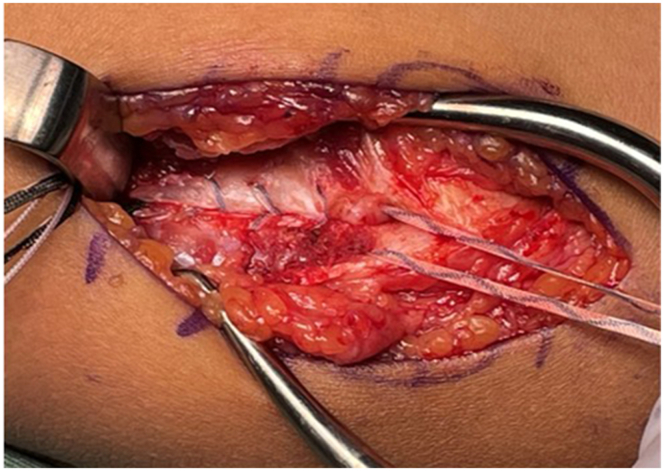
Intraoperative image showing shoelace repair of the split UCL using 2 SutureTape (1.3-mm) limbs from the anchor placed in the sublime tubercle. The white-and-blue–striped tapes are SutureTapes used for the shoelace repair. *UCL*, ulnar collateral ligament.

A 4.75-mm absorbable SwiveLock anchor (Arthrex, Naples, FL, USA), loaded with 2 SutureTapes (1.3 mm; Arthrex, Naples, FL, USA) and one 2-0 FiberWire (Arthrex, Naples, FL, USA) (yielding 6 suture limbs in total), was inserted into the sublime tubercle regardless of tear location (proximal or distal) or the presence of BFs. The 2-0 FiberWire was used for distal ligament repair, whereas 2 of the 4 SutureTape limbs were used to perform a shoelace repair of the split UCL, applying adequate tension to restore ligament continuity (Fig. 3). The remaining 2 SutureTape limbs were used as an IB.

**Figure 4:**
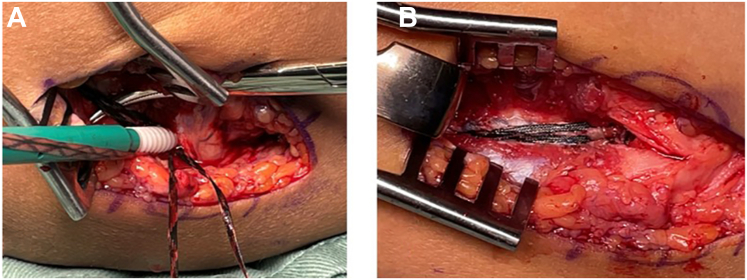
Final fixation using a second SwiveLock anchor at the medial epicondyle. (**A**) Pean clamp inserted under the internal brace to prevent overtensioning. (**B**) Shoelace repair with internal brace, final view. The black SutureTapes (1.3-mm) are used for the internal brace.

All suture limbs were then fixed together using another 4.75-mm absorbable SwiveLock anchor, which was inserted at the inferior margin of the medial epicondyle corresponding to the isometric point of the ligament, with the elbow flexed at 60°. To avoid overtensioning of the IB limbs, a fine Pean clamp was inserted beneath them during fixation (Fig. 4, *A* and *B*).

### Post-operative rehabilitation

The elbow was immobilized with a splint for 1 week following surgery. Starting 1 week post-operatively, a hinged elbow brace was applied, and range of motion exercises were initiated, with both flexion and extension increased by 10° each week (total 20° per week). The brace was removed at 2 months post-operatively. The throwing program was initiated at 3 months post-operatively. RTP was typically targeted at 6 months for position players and 8 months for pitchers, depending on individual recovery.

### Baseline and follow-up patient-reported and return-to-sport outcomes

Clinical outcomes were assessed using the Japan Orthopedic Association Sports (JOA Sports) score, Kerlan–Jobe Orthopedic Clinic (KJOC) Overhead Athletes Shoulder and Elbow score, and Disabilities of the Arm, Shoulder, and Hand (DASH) sports module score. RTP was defined as participation in at least one official game following surgery.

Post-operative competitive level was evaluated using the Conway scale,^9^ where:

• “Excellent” indicated return to the same or higher competitive level for at least 12 months
• “Good” indicated return to a lower competitive level than preinjury
• “Fair” indicated return to recreational play
• “Poor” indicated failure to return to sports

### Statistical analyses

Statistical analyses were performed using R software (version 3.6.3, R Foundation for Statistical Computing, Vienna, Austria). Pre-operative and post-operative clinical outcomes were compared using paired *t*-test and Wilcoxon signed-rank test. Two-group comparisons between the BF and NBF groups for patient characteristics, clinical scores, and time to RTP were conducted using independent samples *t*-test and Mann–Whitney *U* test. Comparisons of UCL injury classification and Conway scale between groups were performed using Pearson chi-square test. A *P* value <.05 was considered statistically significant.

## Results

### Surgical cases and patient characteristics

A total of 34 patients underwent SRIB for UCL injury, of which 18 met the inclusion criteria and were included in this study (Fig. 4). Among them, 8 patients were classified into the BF group (proximal: 6 cases, distal: 2 cases), and 10 were classified into NBF group. Patient characteristics and comparisons between the BF and NBF groups are summarized in Table I. There were no significant differences between the BF and NBF groups in terms of age (17.0 ± 1.2 vs. 16.8 ± 2.3 years; *P* = .37), height (173 ± 0.8 vs. 172 ± 0.6 cm; *P* = .75), weight (71.5 ± 6.1 vs. 73.7 ± 9.9 kg; *P* = .59), body mass index (BMI) (24.7 ± 2.4 vs. 23.7 ± 1.1 kg/m^2^; *P* = .30), competition level (7 high school and 1 college student vs. 9 high school and 1 college student; *P* = .59), or player position (5 pitchers, 2 catchers and 1 infielders vs. 6 pitchers, 1 catchers, 2 infielders, and 1 outfielders; *P* = .66).

**Table I:** Patient characteristics.

Outcome	BF (N = 8)	NBF (N = 10)	*P* value
Age (yr)	17 ± 1.2	16.8 ± 2.3	.37
Height (cm)	173 ± 0.8	172 ± 0.6	.75
Weight (kg)	71.5 ± 6.1	73.7 ± 9.9	.59
BMI (kg/m^2^)	24.7 ± 2.4	23.7 ± 1.1	.3
Level			.59
High school	7	9	
College	1	1	
Position			.66
Pitcher	5	6	
Catcher	2	1	
Infielder	1	2	
Outfielder	0	1	

*BMI*, body mass index; *BF*, bone fragment; *NBF*, no bone fragment.

Regarding the classification of UCL injuries, 5 patients in the BF group and 6 in the NBF group were classified as type 1A, whereas one patient in the BF group and none in the NBF group were classified as type 1B. No cases in either group were classified as types 2A or 2B.

Two patients in the BF group and one in the NBF group were classified as type 3A, whereas no patients in the BF group and 3 in the NBF group were classified as type 3B. There were no significant differences in UCL injury classification between the 2 groups (*P* = .23) (Table II).

**Table II:** UCL injury classification.

Outcome	BF (N = 8)	NBF (N = 10)	*P* value
UCL injury classification			.23
1A	5	6	
1B	1	0	
2A	0	0	
2B	0	0	
3A	2	1	
3B	0	3	

*UCL*, ulnar collateral ligament; *BF*, bone fragment; *NBF*, no bone fragment.

### Pre-operative and post-operative clinical scores

The pre-operative and post-operative clinical outcomes of the 18 cases (pre-operative/post-operative: *P* value) are shown in Table III. Significant improvements were observed in the JOA sports score (30.5/90.5 points: *P* < .0001), DASH sports score (81.2/6.9 points: *P* < .0001), and KJOC score (40.3/85.3 points: *P* < .0001). The follow-up period for each group were: BF 22.8 months and NBF 21.5 months, with no significant difference between the 2 groups (*P* = .77).

**Table III:** Pre-operative and post-operative clinical score.

Outcome	Pre-operative	Post-operative	*P* value
JOA sports score (points)	30.5 ± 18.7	90.5 ± 11.5	<.0001∗
DASH sports score (points)	81.2 ± 15.3	6.9 ± 13.2	<.0001∗
KJOC score (points)	40.3 ± 15.2	85.3 ± 11.4	<.0001∗

*JOA*, Japan Orthopedic Association; *DASH*, disabilities of the arm, shoulder, and hand; *KJOC*, Kerlan–Jobe Orthopedic Clinic.

∗ *P* < .001.

In the BF group, the pre-operative and post-operative clinical outcomes (pre-operative/post-operative: *P* value) showed significant improvements in the JOA sports score (25.8/87.8 points: *P* = .0001), DASH sports score (79.6/7 points: *P* = .007), and KJOC score (39/85.6 points: *P* = .007).

In the NBF group, the pre-operative and post-operative clinical outcomes (pre-operative/post-operative: *P* value) showed significant improvements in the JOA sports score (35/85.8 points: *P* < .0001), DASH sports score (82.5/6.8 points: *P* = .003), and KJOC score (41.5/85.2 points: *P* < .0001).

In the post-operative clinical outcomes between the 2 groups (BF/NBF: *P* value), there were no significant differences in the JOA sports score (87.8/85.8 points: *P* = .73), DASH sports score (7/6.8 points: *P* = .74), or KJOC score (85.6/85.2 points: *P* = .93) (Table IV).

**Table IV:** Pre-operative and post-operative clinical score of BF and NBF.

Outcome	BF (N = 8)	NBF (N = 10)	*P* value (BF vs. NBF)
Follow-up time (mo)	22.8 ± 10.5	21.5 ± 7.3	.77
JOA sports score (points)			
Pre-operative	25.8 ± 19.5	35.0 ± 17.5	.31
Post-operative	87.8 ± 10.9	85.8 ± 12.5	.73
*P* value (pre-operative vs. post-operative)	.0001∗	<.0001∗	
DASH sports score (points)			
Pre-operative	79.6 ± 17.6	82.5 ± 14.0	.71
Post-operative	7.0 ± 10.2	6.8 ± 15.7	.74
*P* value (pre-operative vs. post-operative)	.007†	.003†	
KJOC score (points)			
Pre-operative	39.0 ± 17.8	41.5 ± 13.8	.64
Post-operative	85.6 ± 9.9	85.2 ± 12.9	.93
*P* value (pre-operative vs. post-operative)	.007†	<.0001∗	

*JOA*, Japan Orthopedic Association; *DASH*, disabilities of the arm, shoulder, and hand; *KJOC*, Kerlan–Jobe Orthopedic Clinic; *BF*, bone fragment; *NBF*, no bone fragment.

∗ *P* < .001.

† *P* < .01.

The RTP duration was 7.5 months for the BF group and 8 months for the NBF group, with no significant difference (*P* = .58). Based on the Conway scale, 7 cases in the BF group were classified as excellent, 1 as good, and none as fair or poor, whereas 8 cases in the NBF group were classified as excellent, 2 as good, and none as fair or poor. No significant difference was observed between the 2 groups (*P* = .67) (Table V). These results suggest that favorable clinical outcomes and RTP can be achieved using SRIB regardless of the presence of BFs. No perioperative or post-operative complications were observed with SRIB in either group.

**Table V:** Return-to-sports competitive performance between bone fragment + or –.

Outcome	BF (N = 8)	NBF (N = 10)	*P* value
TRP (mo)	7.5 ± 2.2	8.0 ± 1.6	.58
Conway scale			
Excellent	7	8	.67
Good	1	2	
Fair	0	0	
Poor	0	0	

*TRP*, time to return to play; *BF*, bone fragment; *NBF*, no bone fragment.

## Discussion

To the best of our knowledge, this is the first clinical study to compare short-term clinical outcomes of SRIB in student baseball players with and without chronic avulsion BFs. Our results demonstrated no significant differences in clinical outcomes between patients with and without BFs, suggesting the feasibility of SRIB as a surgical option in such cases.

Since Dr Frank Jobe first reported on UCL reconstruction in 1974, it has become the gold standard for the treatment of UCL injuries.^10^^,^^17^^,^^21^^,^^36^ In contrast, UCL repair was first performed by Norwood et al^24^ in 1981 for athletes with UCL injuries, and they reported satisfactory outcomes. However, a comparative study evaluating post-operative outcomes of reconstruction vs. repair in overhead athletes reported a RTP rate of 68% for reconstruction and 50% for repair. Among professional athletes, the RTP rate was 75% for reconstruction and 29% for repair, indicating superior outcomes with reconstruction.^9^

In 2006, Argo et al^2^ reported performing UCL repair on female athletes, including overhead athletes, with a 94% RTP rate at their preinjury competitive level. Similarly, Savoie et al^31^ reported an RTP rate of 93%. These favorable outcomes were attributed to the inclusion of student athletes, who are believed to exhibit less ligament degeneration compared with professional athletes.^19^^,^^22^^,^^35^ Since then, advancements in surgical techniques, such as the use of IB and more selective surgical indications, have resulted in RTP rates of 87%-96%, which are comparable to those achieved with reconstruction.^1^^,^^12^^,^^13^^,^^25^^,^^29^^,^^30^ Consistent with previous findings, the present study demonstrated favorable clinical outcomes in student baseball players undergoing SRIB, with all patients successfully returning to competitive play.

Among student athletes with UCL injuries, some present with lesions or avulsion of the medial epicondyle that originated around the age of 10 years, leading to valgus instability and the need for surgical intervention.^32^ In such cases, UCL reconstruction is often indicated in the United States.^11^^,^^13^^,^^31^ This is due to shortening of the UCL and degenerative changes around the BF, which make primary repair infeasible.^23^^,^^34^ However, reconstruction typically requires 11.6-16.8 months for RTP,^6^^,^^10^^,^^17^^,^^21^^,^^36^ resulting in the loss of one to 2 competitive seasons.^33^ Moreover, many high school athletes may be hesitant to undergo surgery owing to the prolonged recovery period required before returning to competition.^19^ One of the advantages of UCL repair is that it allows preservation of the native anatomy and sensory function.^1^

Considering these factors, we adopted SRIB for student baseball players seeking early RTP, regardless of the presence of BFs. Uchida et al^35^ reported that in shoelace repair, passing the suture tape through the distal UCL enables the tendon complex—including the UCL and flexor–pronator muscles—to converge at the medial epicondyle, thereby restoring the dynamic stabilizing mechanism. Although Uchida et al^35^ reported the technical feasibility of SRIB for chronic avulsion injuries, no clinical outcome data were presented, limiting its clinical applicability. The present study addresses this gap by directly comparing outcomes with and without BFs. In particular, the shoelace pattern may enhance not only longitudinal but also transverse tensioning of the ligament, which could further enhance medial elbow stability through improved dynamic muscle function, consistent with the role of the flexor–pronator group as described by Hoshika et al^19^ This technique has been considered useful in managing UCL instability associated with Little League elbow. In the present study, SRIB was employed in all cases, and no significant differences were observed between the BF and NBF groups in clinical outcomes, RTP duration, or Conway scale scores.

This study has some limitations. First, the sample size was small. Second, only short-term outcomes were assessed, and long-term follow-up is warranted. Third, no biomechanical analysis of the shoelace repair technique was conducted. In addition, the assessment of ligament quality during surgery was based on the subjective intraoperative judgment of the operating surgeon, which may have introduced selection bias. Furthermore, potential selection bias due to intraoperative conversion to reconstruction may limit the generalizability of our findings. Moreover, the high RTP rate observed in this cohort may partly reflect the inclusion of highly motivated student athletes with relatively mild degeneration, potentially introducing selection bias. Therefore, the present findings should be interpreted as preliminary clinical evidence, and the absence of significant differences between groups should be viewed cautiously given the limited sample size and statistical power.

Taken together, these findings suggest that SRIB may be considered a viable option for carefully selected young athletes with chronic avulsion BFs, although further prospective studies with larger cohorts are warranted to confirm its long-term effectiveness. Future studies incorporating biomechanical validation and larger, more diverse cohorts are warranted to establish the long-term efficacy of SRIB, especially in athletes with advanced degeneration or higher levels of competition. These findings may help refine surgical decision-making in young athletes with UCL injuries, expanding the indications for repair-based techniques beyond traditional boundaries.

## Conclusion

SRIB was performed in student baseball players with and without chronic avulsion BFs, and post-operative outcomes were comparable between the groups. These findings suggest that SRIB may be a viable and safe surgical option in carefully selected young athletes with adequate tissue quality, thereby providing a balance between stability and early RTP.
